# The Langmuir-Blodgett Technique as a Tool for Homeotropic Alignment of Fluorinated Liquid Crystals Mixed with Arachidic Acid

**DOI:** 10.3390/ijms12084923

**Published:** 2011-08-03

**Authors:** Anna Modlińska, Danuta Bauman

**Affiliations:** Faculty of Technical Physics, Poznan University of Technology, 60-965 Poznan, Poland; E-Mail: anna.modlinska@doctorate.put.poznan.pl

**Keywords:** fluoro-substituted liquid crystal, Langmuir film, Langmuir-Blodgett film, Brewster angle microscopy, electronic absorption

## Abstract

Some fluoro-substituted liquid crystals mixed with arachidic acid in monolayers formed at air-liquid (Langmuir films) and air-solid substrate (Langmuir-Blodgett films) interfaces were investigated. Molecular organization in Langmuir films was determined on the basis of the analysis of the shape of the surface pressure-mean molecular area isotherm and observations made by means of a Brewster angle microscope. It was found that in the compression process the liquid crystal molecules are pushed out towards the top of the first monolayer being in direct contact with the subphase. Langmuir films were transferred onto the quartz substrates at various surface pressures and mono- and multilayered Langmuir-Blodgett films were obtained. The films were characterized using electronic absorption measurements. The conditions for obtaining the homeotropic orientation of the liquid crystal molecules were determined.

## 1. Introduction

Liquid crystal displays (LCDs) are still the most dominant displays, which are widely used commercially. However, to stay on the market and be competitive with other types of displays, such as plasma display panels (PDPs), field emission displays (FEDs), surface electron-emitter displays (SEDs), organic light emitting diodes (OLEDs), *etc.*, their parameters must be continually improved. This relates mainly to their disadvantages, such as the relatively narrow viewing angle, long reaction times, the problem of obtaining a deep blackness. The parameters of liquid crystal displays can be improved using new effects and novel liquid crystal materials with pre-designed appropriate physical properties.

In the last decade, fluorinated liquid crystals have attracted much attention from researchers. It was found that the replacement of one or more hydrogen atoms by fluorine atoms in the molecular structure leads to liquid crystalline compounds characterized by specific, unique properties [1–3]. The biggest advantages of these type of compounds are very stable, relatively broad mesophase range, low viscosity, low conductivity and low threshold voltage. Therefore, these materials are suitable for use as components of commercial mixtures for twisted nematic (TN) or super twisted nematic (STN) liquid crystal displays, and especially those that are controlled by using thin-film transistors (TFT) [4,5]. Mesogenic molecules, in which fluorine atoms are substituted in the lateral position, are particularly interesting. Such substitution may indeed lead to obtain liquid crystalline compounds with negative dielectric anisotropy. The demand for this type of liquid crystals grew at the time of development and use of liquid crystal displays working in vertical alignment (VA) technology [5]. In comparison to the most popular TN LCDs, VA-type displays are characterized by a much wider, symmetrical viewing angle, enhanced picture quality and contrast, less visible pixel structure. They are also easier to control through an electric field, due to their low anchoring energy. Of all available types of displays, VA LCDs are considered to be the best color mapping. However, it is very difficult to obtain a good, uniform initial arrangement of liquid crystal molecules. The initial molecules alignment in TN-type displays has to be planar, and it is relatively easy to obtain and is now well controlled. VA-type displays require preliminary homeotropic orientation of molecules. There are several techniques to obtain such an arrangement [6], most of them need the use of a suitable surfactant. However, it is not always possible to get good quality molecule alignment. An alternative way to obtain initial homeotropic orientation of liquid crystal molecules is the utilization of the technique based on deposition onto a solid substrate a Langmuir-Blodgett (LB) film [7] from an appropriate fatty acid, acting as a layer inducing orientation (“command layer”) [8,9]. Using this technique, one can impose required alignment of liquid crystal molecules (perpendicularly to the surface or at a small, well-defined angle).

The use of LB technique to obtain a layer orienting nematic liquid crystal molecules was proposed by Komitov *et al.* [8], who used to this end lecithin and its derivatives as well as stearic acid. In subsequent papers [10,11], they describe how to obtain a uniform homeotropic alignment of liquid crystal molecules by LB films formed from other fatty acids and their mixtures. However, Kuehnau *et al.* [12] proposed LB films formed of a mixture of fatty acids and the liquid crystal 4-*n*-pentyl-4′-cyanobiphenyl (5CB) to use as a layer inducing the orientation of molecules. A similar method was applied by Collins *et al.* [13], who for homeotropic alignment of molecules of technologically important liquid crystalline mixtures with negative dielectric anisotropy used the LB film formed from this mixture with arachidic acid.

In this paper we describe the behavior of the mixtures of some fluoro-substituted liquid crystals with arachidic acid in monolayers formed at air-liquid (Langmuir films) and air-solid substrate (LB films) interfaces. We tried to determine the best conditions for obtaining the uniform homeotropic alignment of fluorinated liquid crystal molecules by using Langmuir-Blodgett technique.

## 2. Materials and Methods

**Figure 1.**
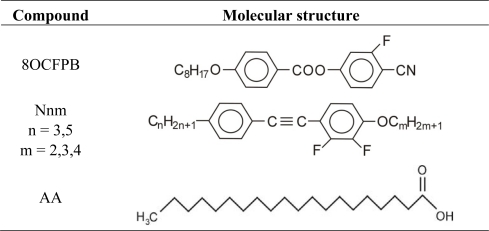
Molecular structure of the compounds studied.

The molecular structure of the compounds investigated is given in Figure 1. The liquid crystals were synthesized and chromatographically purified in Prof. R. Dąbrowski’s Laboratory at the Institute of Chemistry, Military University of Technology in Warsaw, Poland. They all possess only the nematic (N) phase between the crystalline (Cr) and isotropic (I) states. The liquid crystal 8OCFPB (4-cyano-3-fluorophenyl 4′-n-octyloxybenzoate) is substituted in the lateral position with the fluorine atom, but due to the presence of the strongly polar cyano-group, its dielectric anisotropy is positive. Six other liquid crystals (Nnm), having two fluorine atoms in lateral positions, are characterized by a negative dielectric anisotropy. Arachidic acid (AA) with a quoted purity of >99% was purchased from Sigma-Aldrich and used as received.

Langmuir and Langmuir-Blodgett films were formed in a Minitrough 2 (KSV Instruments Ltd., Espoo, Finland). This trough was equipped with two barriers for monolayer compression. We used the pure water or calcium-containing buffer solution as the subphase. The water was deionized and purified to a final resistivity of 18.2 MΩ·cm by a Milli-Q system (Millipore Corporation, Wien, Austria). The buffer was composed of 10^−3^ mol·dm^−3^ CaCl_2_ dissolved in a purified water. A constant subphase temperature was maintained by a cooling circulator and kept constant at 21 °C. Both liquid crystal materials and AA were dissolved in chloroform (Uvasol) at a concentration of 0.1 mM in order to obtain stock solutions. The concentrations of solutions were confirmed spectroscopically. The mixed solutions of the liquid crystal/AA of selected molar fraction X_M_ were prepared at room temperature shortly before spreading at the air-water interface. The surface of the subphase was carefully purified using an aspirator and then an appropriate amount (from 40 to 120 μL) of the mixed solution was spread drop by drop from a microlitre syringe (Hamilton, Birmingham, England). The chloroform was allowed to evaporate for 15 min after spreading.

The most basic characterization of Langmuir films is the measurement of the surface pressure, π, *versus* the average area available for one molecule, A, at constant temperature (π-A isotherm) [7,14]. For monolayers, π is defined as the surface tension of pure subphase minus the surface tension of the subphase-monolayer system. In our experiment the monolayer was compressed symmetrically from both sides at a barrier motion speed of 5 mm·min^−1^ (approximately 2 × 10^−7^ nm^2^·molecule^−1^·s^−1^), while the surface pressure was monitored by a Wilhelmy plate balance with an accuracy of ±0.1 mN/m. All measurements were repeated on fresh subphases three to five times to confirm reproducibility. Standard trough cleaning procedure was adopted between measurements.

The morphology of the Langmuir films was visualized by means of a Brewster angle microscope (BAM). The instrument we used is based on Hoenig and Moebius setup [15] and was built in our laboratory. The image features were observed with a lateral resolution of ≈5 μm.

Polished quartz plates (35 × 10 × 1 mm^3^) were used as the solid substrates with hydrophilic surfaces for LB film fabrication. In order to obtain hydrophobic surface, special procedures, described in [16], were used. The vertical dipping method was used and dipping rate was 5 mm/min. Langmuir films were deposited onto quartz plates at the appropriate surface pressures determined from π-A isotherm for given mixture. The dipping stroke was 20 mm. The mono- and Y-type multilayered LB films were prepared. In the case of multilayers after each emersion, the substrate was dried with dry air for 20 min before the next immersion. One of the key indices used to determine the deposition quality is the transfer ratio, TR, which was calculated as the ratio of the decrease in the subphase area to the area on the substrate coated by the layer.

The absorption spectra of LB films were recorded in the UV region by means of a spectrophotometer CARY 400. The incident light beam was normal to the substrate surface.

## 3. Results and Discussion

### 3.1. Surface Pressure-Mean Molecular Area Isotherms of Langmuir Films

The liquid crystal 8OCFPB is able to form compressible and stable Langmuir films, which can be transferred onto the solid substrates making LB films. The characterization of both kinds of films of this liquid crystal is described in [17]. However, after spreading solutions of the liquid crystals Nnm on the subphase in the trough, we observed large patches of the liquid crystal on the water or CaCl_2_ solution, and the surface pressure did not rise at the compression process. This means that none of the Nnm compounds can produce compressible monolayer at the air-liquid interface. Therefore, in order to study the properties of these liquid crystals in Langmuir and LB films it was necessary to use an arachidic acid as the supporting matrix. The liquid crystals were mixed with AA at various concentrations. It was ascertained that, up to the liquid crystal molar fraction X_M_ = 0.6, the compression of the monolayers formed of liquid crystal/AA mixtures was possible and stable Langmuir films were obtained.

**Figure 2.**
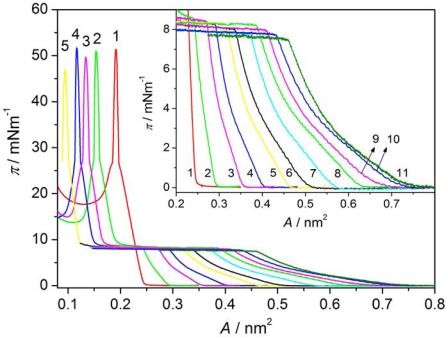
Surface pressure-mean molecular area isotherm of Langmuir films of arachidic acid (AA) (**1**), 4-cyano-3-fluorophenyl 4′-n-octyloxybenzoate (8OCFPB) (**11**), and 8OCFPB/AA mixtures at X_M_ of the liquid crystal: 0.1 (**2**), 0.2 (**3**), 0.3 (**4**), 0.4 (**5**), 0.5 (**6**), 0.6 (**7**), 0.7 (**8**), 0.8 (**9**), and 0.9 (**10**). The insert contains enlarged isotherms in the vicinity of the liquid crystal collapse point.

**Figure 3.**
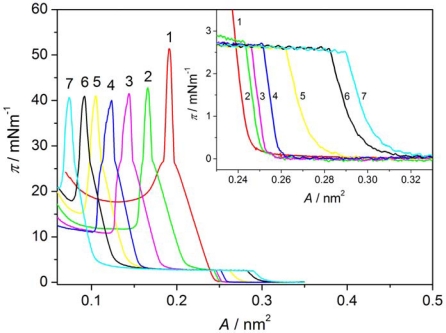
Surface pressure-mean molecular area isotherm of Langmuir films of AA (**1**) and N54/AA mixtures at X_M_ of the liquid crystal: 0.1 (**2**), 0.2 (**3**), 0.3 (**4**), 0.4 (**5**), 0.5 (**6**), and 0.6 (**7**). Insert contains enlarged isotherms in the vicinity of the liquid crystal collapse point.

Figure 2 shows π-A isotherms of Langmuir films of AA (curve 1) and the liquid crystal 8OCFPB (curve 11) as well as of their mixtures at various X_M_ of the liquid crystal (curves 2–10) on pure water as the subphase. The isotherm of AA on the water as well as that obtained on CaCl_2_ solution are in good agreement with the ones given in literature [18,19]. The representative isotherms of monolayers on the water for liquid crystals Nnm mixed with AA are presented in Figure 3 for N54/AA mixtures (curves 2–7). It is seen that when the liquid crystal is added to AA, the shape of π-A isotherm changes. For mixed Langmuir films the plateau region, characteristic for many thermotropic liquid crystals of rod-like shaped (calamitic) molecules [17,20–25] appears. Such a plateau region has already been observed by Xue *et al*. [20], and subsequently by Friedenberg *et al*. [21] and de Mul and Mann [22], who investigated the behavior of the liquid crystal 4-octyl-4′-cyano-biphenyl (8CB) in the Langmuir film. Xue *et al*. [20] postulated the existence of an interdigitated bilayer on top of a monolayer (trilayer) adjacent to the interface in this area region. According to de Mul and Mann [22], rigid molecular cores in the bilayer are oriented perpendicularly to the subphase surface, like in the liquid crystalline smectic A phase. Our previous investigations of many thermotropic liquid crystals with different molecular structure of calamitic molecules [17,24,25] indicated that the plateau region is characteristic for most liquid crystals, which are able to create compressible monolayer at the gas-liquid interface. However, analysis of the π-A isotherm shape, supported by BAM images [17,24] and surface potential measurements [17], showed that there exists some correlation between the organization of molecules in the Langmuir film and their ability to form the appropriate mesophase in the bulk. Whereas smectogenic molecules in the plateau region create the interdigitated bilayer, the nematogenic molecules form 3D droplet-like domains with nematic-like arrangement. Thus, the appearance of the plateau is indicative of incorporation of the liquid crystal molecules in a monolayer, even in the case of the liquid crystal N54, which does not create itself the compressible monolayer at the subphase surface. Similar results were obtained for all other liquid crystals from Nnm series, although the plateau region occurred at various surface pressures. These results are also in agreement with observations of Collins *et al*. [13] for the liquid crystal Merck N4 mixed with AA.

The characteristic data of π-A isotherms for the Langmuir films of the liquid crystal/AA mixtures on the water and CaCl_2_ solution are gathered in Tables 1–3 and 4–6, respectively. In the tables, the following data are presented: A_0_–the value of the area at which π starts to rise, A_C_–the value of the area at the collapse point, and π_C_–the value of the collapse pressure. The collapse point is recognized here as the point in the isotherm where the ratio ∂π/∂A starts to decrease due to a next phase transition. Thus, for the most mixed Langmuir films, two regions with the rapid increase of π and two collapse points can be distinguished. Two collapse points were previously reported in the literature for monolayers formed by mixtures of two components, each of whom has a different shape of π-A isotherm [12,13,25–27]. In the case of the liquid crystal/AA mixtures under investigation the first collapse point observed at the beginning of the plateau region can be ascribed to the liquid crystal phase transition and the second one can be related to the phase transition of AA.

More information about the Langmuir films of the liquid crystal/AA mixtures gives the compressibility coefficient, C_S_, defined as follows:

(1) $$C_{S}=-\frac{1}{A}\frac{dA}{d\pi }$$

Taking into account only the fairly straight section of the isotherm, following Ras *et al*. [28], we can determine the “apparent” compressibility, C_S_′:

(2) $$C_{S}{}^{\prime }=-\frac{1}{A_{1}}\frac{A_{2}-A_{1}}{\pi_{2}-\pi_{1}}$$

where A_1_ and A_2_ correspond to the area per molecule at surface pressures π_1_ and π_2_, respectively. The “apparent” compressibility coefficients C_S_′ are also presented in Tables 1–6. In our calculations, π_1_ and π_2_ were selected at the beginning and the end of the linear regions on the π-A isotherm corresponding to the liquid crystal and AA.

The results obtained for the Langmuir films on both kinds of subphases do not significantly differ. Solely in the case of the liquid crystal N32, addition of CaCl_2_ to the water facilitates the formation of the monolayer with higher content of the liquid crystal. From the data presented in the above tables it is seen that only the liquid crystal N52 has plateau region at the value of the surface pressure similar to that of the liquid crystal 8OCFPB. For other liquid crystals from the Nnm series, the plateau occurs at significantly lower π. The surface pressures at two collapse points are almost independent of the mixture composition for all the liquid crystals under investigation. However, the compressibility in most cases increases with increasing liquid crystal content. It can be seen very clearly for the liquid crystal 8OCFPB, both in the region related to the liquid crystal and to AA. This is understandable taking into account the fact that the molecules of 8OCFPB, similarly as those of other calamitic liquid crystals, are tilted at a very large angle with respect to the normal to the subphase surface and therefore they are loosely packed in the monolayer [17]. The addition of AA improves the packing density of the liquid crystal molecules and as a result the rigidity of the Langmuir film increases. The change of the rigidity can be tracked on the basis of the isotherm slope (∂π/∂A). From Figures 2 and 3 as well as from the data presented in Tables 1–6, it follows that the slope of the π-A isotherm for 8OCFPB/AA mixture increases with the rise of AA content. This means that AA molecules are able to “stiffen” molecules of this liquid crystal. In the case of Nnm/AA mixtures, the changes of the compressibility coefficient are not very regular, but the tendency to an increase of C_S_′ value with increasing X_M_ is observed, especially for monolayers formed on the buffer with CaCl_2_. Small differences between behavior of the Langmuir films on both kinds of subphases can be connected with the change of the electrostatic interaction in the absence and presence of Ca^2+^ ions [19,28].

The mean molecular area at the collapse point for AA corresponds almost to the cross section of the aliphatic chain (≈0.2 nm^2^ [8], HyperChem program calculations), indicating an average vertical arrangement of the chains with respect to the subphase surface in the compressed Langmuir film formed of AA itself. The in-plane cross section of the liquid crystal molecule, laterally substituted with fluorine atom (or atoms), is about 0.3 nm^2^ (HyperChem program calculations). Thus, at compression of the mixed Langmuir films below A = 0.2 nm^2^ (already at the X_M_ = 0.1 for the liquid crystals from Nnm series), the average area per molecule is too small for even the most dense packing of molecules in a monolayer. This suggests that some molecules are pushed out from the subphase surface and create three-dimensional (3D) objects on the top of the monolayer adjacent to the interface.

To study quantitatively the molecular organization of the mixed monolayers at the air-liquid interface, the miscibility or the phase separation of two components should be determined. Following Gaines [14] we can made it on the basis of the shape of π-A isotherms for various X_M_ using the excess criterion and the surface phase rule. Let us define the excess of the average area per molecule, A_E_, at given surface pressure as follows:

(3) $$A_{E}\;=\;A_{12}-(X_{M1}A_{1}+X_{M2}A_{2})$$

where A_12_ is the average molecular area in the two-component film, X_M1_ and X_M2_ are the molar fractions of two components, and A_1_ and A_2_ are the molecular areas of two single-component films at the same π.

**Figure 4.**
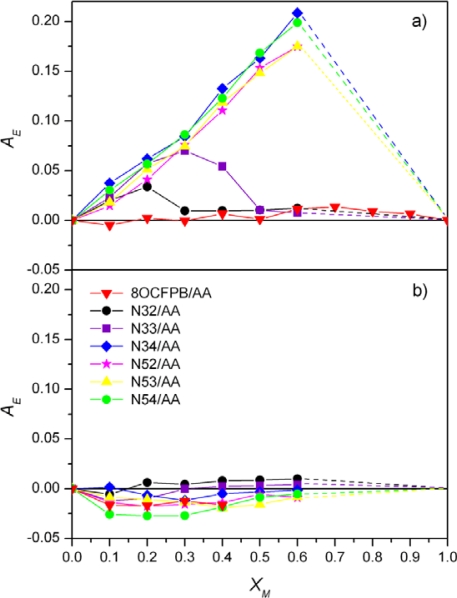
Excess area as a function of the molar fraction of the liquid crystal for 8OCFPB and Nnms at π below the plateau region (**a**) and at π = 20 mN/m (**b**).

If A_E_ is equal to zero, the average area per molecule follows the additivity rule, A_12_ = X_M1_A_1_ + X_M2_A_2_, which means that in the mixture ideal mixing or complete immiscibility occurs. Deviation from zero, either positive or negative, indicates miscibility and non-ideal behavior [14,29]. The excess area per molecule as a function of X_M_ for mixed Langmuir films at the surface pressure below the first collapse point and at π = 20 mN/m is plotted in Figure 4(a) and 4(b), respectively. The data for monolayers formed on pure water subphase are presented and they are very similar to those obtained on the buffer. No deviation from the additivity rule at both surface pressures is observed only for the mixtures of AA with 8OCFPB. In the case of the liquid crystals Nnm the values of A_E_ > 0 at low π were obtained, with exception of N32/AA and N33/AA mixtures at higher X_M_. The positive deviation from the additivity rule is characteristic for miscible components with repulsive intermolecular interactions. At π = 20 mN/m the additivity rule for all the Nnm/AA mixtures is fulfilled. However, in order to recognize if we are dealing with an ideal mixing or a phase separation, the information from the surface phase rule [14] should be taken into account additionally. This rule states that if the components are miscible, the π_C_ value should change with the mixture composition. Thus, the dependence of the π_C_ value on the composition of the Langmuir films formed from the liquid crystal/AA mixtures seen in Tables 1–6 indicates that the molecules of 8OCFPB and AA are immiscible, both at low and high surface pressures. In the case of Nnm/AA mixtures at most partial miscibility of molecules at low π can be postulated. This results from the fact that, although the additivity rule is not fulfilled, π_C_ remains constant. However, in the compressed monolayers the phase separation of two components occurs. The difference between A_E_ values in various stages of the Langmuir film compression seems to suggest that reducing the available area, the molecules of the liquid crystal are pushed out towards the top of the monolayer.

### 3.2. Brewster Angle Microscopy Images

**Figure 5.**
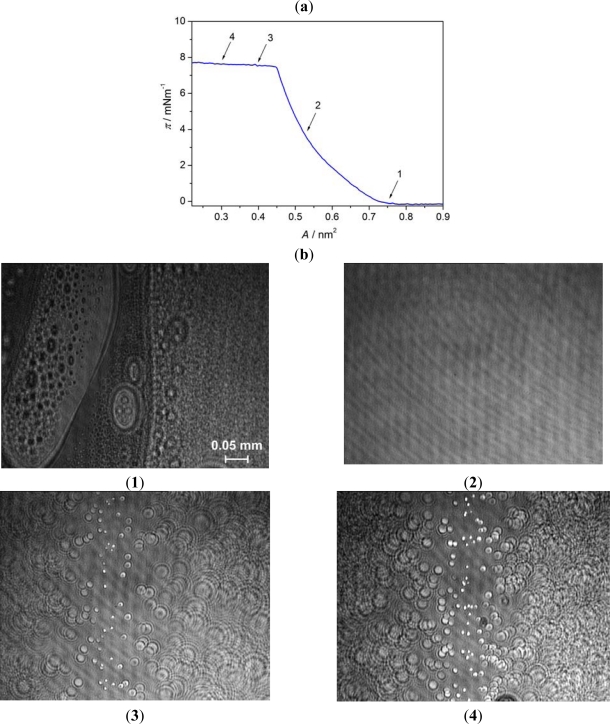
Surface pressure-mean molecular area isotherm (**a**) and BAM images (**b**) of Langmuir film of 8OCFPB at A = 0.75 nm^2^ (**1**), 0.53 nm^2^ (**2**), 0.40 nm^2^ (**3**), and 0.30 nm^2^ (**4**). The arrows indicate the compression stages at which BAM images were recorded.

Brewster angle microscopy gives possibility for direct observation of the morphology of Langmuir films. Imaging of the surface film was performed at different surface pressures during a slow continuous compression of the monolayer formed on the water. Figure 5 illustrates the change of the textures of 8OCFPB monolayer during compression process. The marks on π-A isotherm (Figure 5(a)) indicate the compression stages at which BAM images were recorded. In the region of coexistence of the gas and liquid phases (Figure 5(b):1), we observe condensed monolayer islands in equilibrium with a foam-like structure. As the surface pressure increases (A = 0.70 − 0.45 nm^2^), the islands pack together into a completely compressed monolayer, giving a homogeneous picture (Figure 5(b):2). After the collapse point, when the surface pressure remains constant (the plateau region), we can observe the appearance of the domains of quite high brightness surrounded by interference rings (Figure 5(b):3). With the decrease of the A value, the number of the domains rises (Figure 5(b):4).

**Figure 6.**
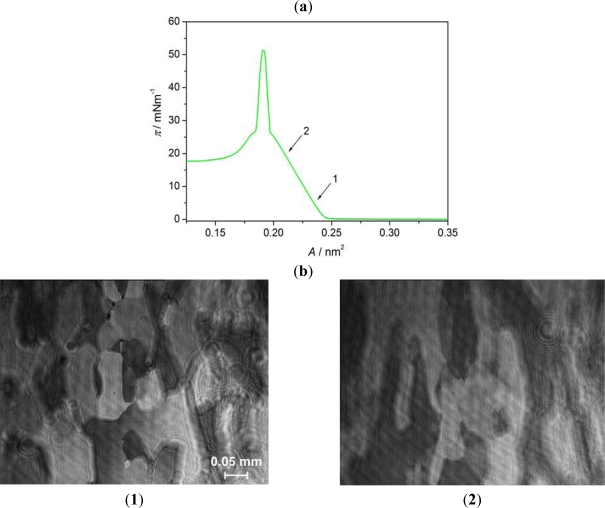
Surface pressure-mean molecular area isotherm (**a**), and BAM images (**b**) of Langmuir film of AA at A = 0.23 nm^2^ (**1**) and 0.21 nm^2^ (**2**). The arrows indicate the compression stages at which BAM images were recorded.

However, they are not in collision and do not join together. The images obtained are typical for those of nematogenic liquid crystals, which were presented in references [17,24]. Thus, we are dealing here with 3D droplet-like domains. BAM images acquired for AA below the collapse point are shown in Figure 6. As in this stage of the monolayer formation, the AA molecules are tilted away from the surface normal the monolayer looks like a mosaic made of domains with different grey level, each corresponding to various direction of molecules tilt [30].

**Figure 7.**
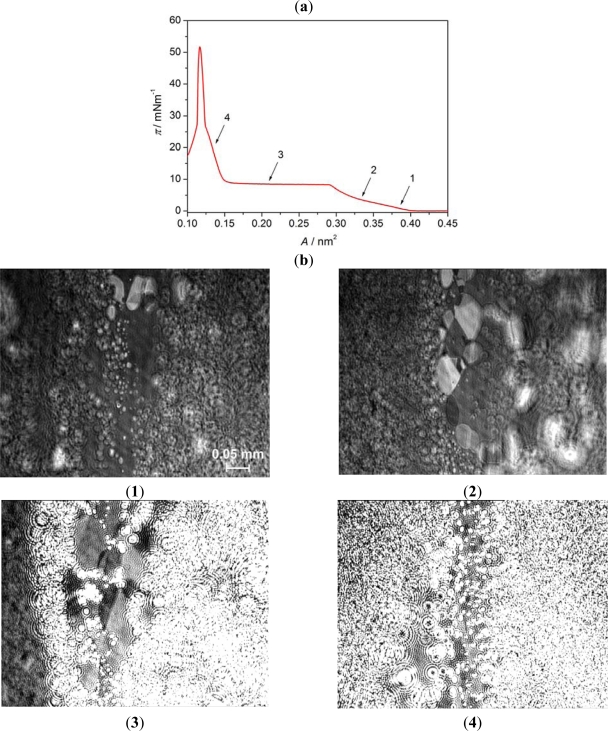
Surface pressure-mean molecular area isotherm (**a**) and BAM images (**b**) of mixed Langmuir film of 8OCFPB/AA (X_M_ = 0.3) at A = 0.38 nm^2^ (**1**), 0.33 nm^2^ (**2**), 0.21 nm^2^ (**3**), and 0.13 nm^2^ (**4**). The arrows indicate the compression stages at which BAM images were recorded.

Figure 7 presents BAM images of the monolayer of 8OCFPB/AA mixture at X_M_ = 0.3. As the surface pressure starts to rise (Figure 7(b):1) two kinds of domains, characteristic for AA and 8OCFPB, appear. At further film compression the monolayer surface is still inhomogeneous with coexisting regions containing separately AA and liquid crystal molecules (Figure 7(b):2). This is in very good agreement with our previous suggestion that molecules of AA and 8OCFPB in the Langmuir film are immiscible. In the plateau region we observe the creation of domains with interference rings, characteristic for nematic liquid crystal (Figure 7(b):3). The detailed analysis of the image seems to indicate that the molecules of 8OCFPB are pushed over the first monolayer being in direct contact with the subphase. This monolayer consists mostly of AA molecules, which, as being more amphiphilic than liquid crystal molecules, remain on the subphase surface. The compression below A = 0.15 nm^2^ (Figure 7(b):4) causes the rapid increase of the number of the liquid crystal domains which gradually cover the whole top of the film.

**Figure 8.**
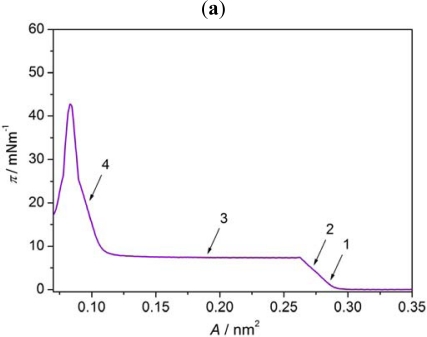

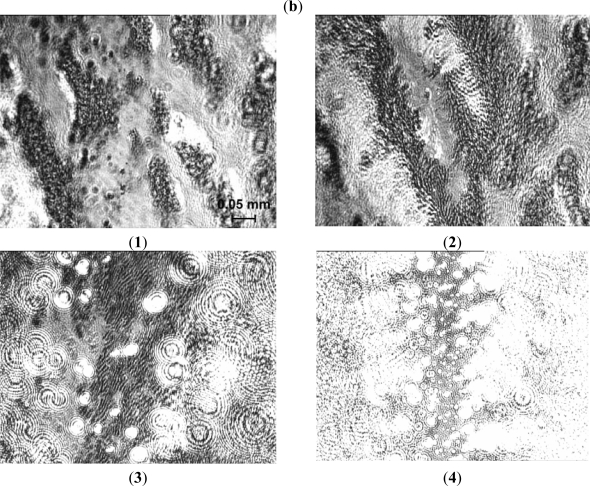
Surface pressure-mean molecular area isotherm (**a**), and BAM images (**b**) of mixed Langmuir film of N52/AA (X_M_ = 0.5) at A = 0.28 nm^2^ (**1**), 0.27 nm^2^ (**2**), 0.19 nm^2^ (**3**), and 0.09 nm^2^ (**4**). The arrows indicate the compression stages at which BAM images were recorded.

**Figure 9.**
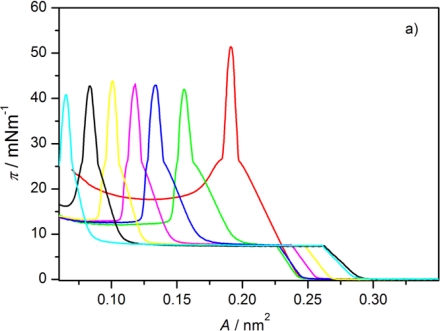

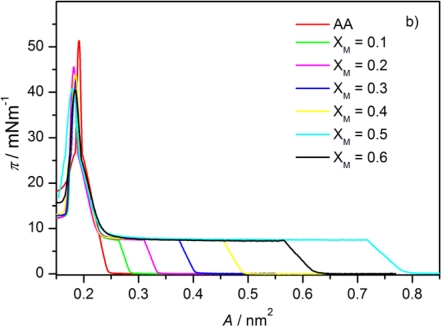
(**a**) Surface pressure-mean molecular area isotherm of mixed Langmuir film of N52/AA at various X_M_ of the liquid crystal; (**b**) The same isotherms as in Figure (a) but only the number of AA molecules was taken into account in the calculation of the molecular area.

**Figure 10.**
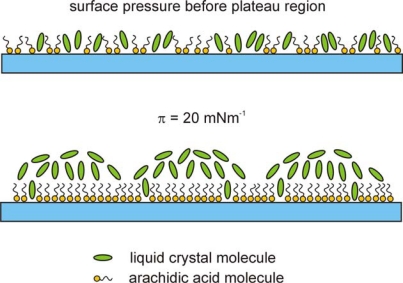
Proposed model of molecular organization in the Langmuir film of the liquid crystal/AA mixture at low and high surface pressures.

BAM images of the monolayers of Nnm/AA mixtures at low surface pressure differ significantly from those obtained for 8OCFPB/AA mixture, which is illustrated in Figure 8 for N52/AA mixture at X_M_ = 0.5, as an example. At the area corresponding to the onset of the π rise, the monolayer appears highly textured with irregular large regions of different grey levels (Figure 8(b):1), which do not significantly change up to the collapse point (Figure 8(b):2). The image of each region resembles orange-peel, similar to previous observations for the liquid crystal 4-pentyl-4′-cyano-*p*-terphenyl (5CT) in the reference [25]. No regions characteristic for AA or a liquid crystal can be unequivocally distinguished, even at smaller Nnm content in the mixture. Behind the collapse point, in the plateau region, on the orange-peel background, the domains with the interference rings begin to appear (Figure 8(b):3) and their number increases with the area reduction (Figure 8(b):4), as in the case of the film of 8OCFPB/AA mixture. This confirms the suggestion drawn on the basis of the dependence of the excess area on the surface pressure (Figure 4) that at higher surface pressure the liquid crystal are removed from the film and accumulate on the top of the monolayer consisting mostly of AA molecules. Such removal of the liquid crystal molecules from the compressed monolayer can be clearly recognizable by comparing of Figures 9(a) with 9(b). Figure 9(a) shows π-A isotherms of N52/AA mixtures on the water surface recorded at various X_M_. Isotherms presented in Figure 9(b) are recalculated: in calculation of the molecular area, only the number of AA molecules was taken into account. It is seen that at the area below 0.2 nm^2^, the run of all the isotherms is nearly identical, independently of the N52 content. Similar results were obtained for all the liquid crystal/AA mixtures under investigation and they mean that both 8OCFPB and Nnm molecules are displaced out from the compressed AA monolayer. The suggested alignment of AA and liquid crystal molecules in the Langmuir film at low and high surface pressures is depicted schematically in Figure 10.

### 3.3. Absorption Spectra of LB Films

**Figure 11.**
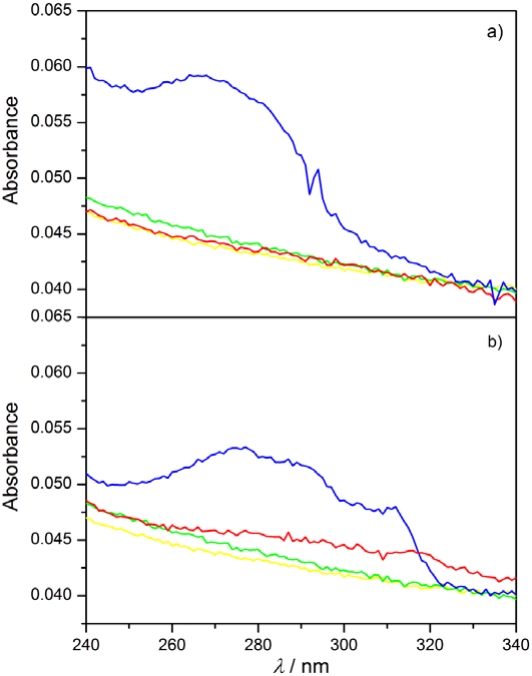
UV absorption spectra of monolayered Langmuir-Blodgett (LB) films of 8OCFPB/AA (**a**), and N54/AA (**b**) mixtures at X_M_ of the liquid crystal equal to 0.3 deposited below plateau region (blue curve) and at π = 20 mN/m (red curve). The yellow and green curves correspond to absorption spectrum of AA in LB film deposited at lower and higher pressures, respectively.

**Figure 12.**
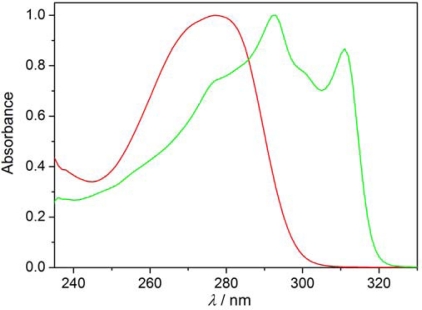
UV absorption spectra of 8OCFPB (red curve) and N53 (green curve) dissolved in chloroform at X_M_ = 4 × 10^−6^.

The monolayers floating on the water were transferred on the quartz substrates at two different surface pressures: before the plateau region and at a π = 20 mN/m. The transfer took place in the upward movement of the substrate at a concave meniscus. For the monolayered LB films obtained with the transfer ratio TR ≈ 1 the absorption spectra were recorded. Typical results are summarized in Figure 11. The absorption spectra for 8OCFPB/AA mixture transferred from pure water (Figure 11(a)) and N54/AA mixture transferred from CaCl_2_ solution (Figure 11(b)) at two surface pressures are presented there. For comparison, in Figure 11 we show additionally the spectra of LB films of AA transferred at appropriate conditions, and in Figure 12 the spectra of 8OCFPB and N54 dissolved in chloroform at X_M_ = 4 × 10^−6^. The absorption band of the liquid crystal is seen distinctly in the LB films deposited at low surface pressure which means that the liquid crystal molecules remain incorporated in the monolayer during the transfer. For the films obtained at π = 20 mN/m, a strong decrease or even complete lack of absorbance is observed.

It can be assumed that the absorption transition moment of rod-shaped liquid crystal molecules is directed along the long molecular axis [31], and the absorption intensity is related to the projection of this moment on the surface. Therefore, after the transfer of the floating monolayer of the liquid crystal/AA mixture at low surface pressure (π = 6 mN/m for 8OCFPB and 2 mN/m for N54) the molecules are not densely packed and as a result the long axes of the liquid crystal molecules are tilted with respect to the quartz surface, which is reflected in distinct absorbance of the LB film. In our previous paper [32] we supposed that at π = 20 mN/m, when the molecules are strongly compressed, they align perfectly perpendicular and the projection of the absorption transition moment on the surface decreases, leading to the absorbance diminishing. However, the analysis of the π-A isotherms and BAM images, described in previous section, contradicts this assumption. Now it seems that, at the transfer of the strongly compressed monolayer, only the first layer consisting mostly of AA molecules covers the quartz substrate, while the liquid crystal molecules being on the top of this layer slide down and remain on the subphase surface.

**Figure 13.**
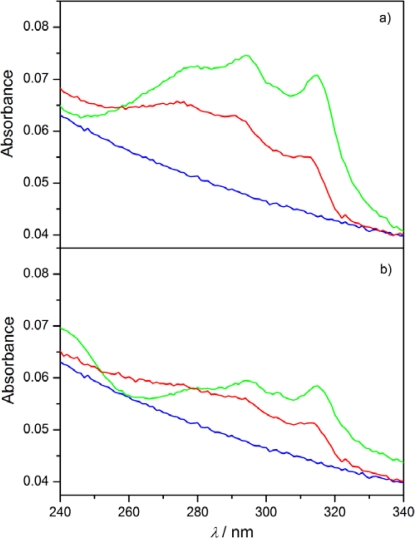
UV absorption spectra of N53/AA mixture at XM = 0.5 in Y-type LB films of multilayer of liquid crystal/AA mixture on hydrophilic quartz substrate (**a**), and of multilayer of liquid crystal/AA mixture on hydrophilic quartz substrate first covered by multilayer of AA (**b**). Blue curve corresponds to absorption spectrum of multilayer of AA. Red and green curves correspond to absorption spectra of mixture N53/AA at π = 2 mNm^−1^ and π = 20 mNm^−1^, respectively.

Previously, Komitov *et al.* [8] found that in order to use the LB film of amphiphilic molecules to homeotropic alignment of liquid crystal molecules it is better to fabricate multilayered films as they are more stable with time. Therefore, in the next step we made the multilayered LB films of three different types: (i) multilayers of the liquid crystal/AA mixtures on hydrophilic substrates; (ii) multilayers of the liquid crystal/AA mixtures on hydrophobic substrates; and (iii) multilayers of the liquid crystal/AA mixtures on hydrophilic substrates first covered by the multilayer of AA deposited at π = 20 mN/m. Multilayered LB films were fabricated only for the liquid crystals with Δɛ < 0 and only using pure water as the subphase (the attempt to make multilayers by taking the CaCl_2_ solution as a subphase failed). Moreover, it turned out that TR value at repeated deposition of the Langmuir film on the hydrophobic substrate at π below the collapse point was very low and in following the LB films obtained in such a way were not taken into account. Figure 13(a) and 13(b) shows the absorption spectra of N53/AA mixture at X_M_ = 0.5 in the LB films of (i) and (iii) types, respectively.

It was not very easy to obtain multilayered LB film of type (i) transferring the floating monolayer at low surface pressure. The transfer was only possible in upstroke, while at the immersion operation, the part of the layer slid down despite the convex meniscus. Therefore, in order to obtain the LB film with the total TR ≥ 2 it was necessary to make 6 dipping cycles (the total transfer was calculated as the ratio of the total decrease of the surface area of the film at the air-water interface to the product of the total surface area of the substrate covered by the film and the number of cycles). At π = 20 mN/m only 2 dipping cycles were made and the deposition took place in the first upstroke of the substrate and next both at downward and upward movements with TR ≤ 1. In this way 3 layers were deposited.

Covering the quartz substrate by AA the hydrophobic surface was obtained, thus the transfer of the monolayer from the water surface was possible both at immersion and emersion with TR ≈ 1 in both directions and as a result two-layered LB film was formed. Comparing Figure 13 with Figure 11 it is seen that the absorption band is now clearly observable not only for the LB films deposited at the low surface pressure, but also for those obtained by transferring at π = 20 mN/m. In the absorption band, three maxima, at λ ≈ 275, 290 and 310 nm, can be distinguished in all of the LB films formed of the liquid crystals Nnm. For Nnm dissolved in chloroform (Figure 12), the maxima occur at λ = 277, 291 and 311 nm and the halfbandwidth δ is equal to (5820 ± 10) cm^−1^. The rough estimation of the bandwidths for the LB films of Nnm/AA mixtures gives the value δ = (6500 ± 500) cm^−1^. Similarly, the lack of the maximum shift with simultaneous significant broadening of the absorption band in the LB films as compared to the appropriate band in diluted solution was found previously for some rod-shaped thermotropic liquid crystals [17]. This was explained in the term of the tendency of the liquid crystal molecules to create the self-aggregates of I-type [33] in the LB films. However, the surface interaction cannot also be excluded.

The appearance of the absorption band in the multilayered LB films deposited at high surface pressure is in the agreement with the results obtained by Collins *et al.* [13]. It seems that after the first emersion of the quartz plate, when the substrate surface becomes hydrophobic and at the next dipping the convex meniscus is created, the liquid crystal molecules from the top of the Langmuir films are able to join their hydrophobic chains to the surface and hence they are present in the LB film. A similar situation occurs when the substrate is first covered by AA layers. However, the molecules are loosely packed and are tilted with respect to the surface, resulting in a relatively high absorbance. Smaller absorption intensities observed for the LB films presented in Figure 13(b) seem, however, to indicate that when the solid substrate surface is previously coated by AA layers the better homeotropic alignment of molecules can be achieved in the films made both at low and high surface pressures.

## 4. Conclusions and Perspectives

In this paper, the molecular organization of seven fluorinated liquid crystals mixed with arachidic acid in Langmuir and Langmuir-Blodgett films were studied. For the Langmuir films the surface pressure-mean molecular area isotherms were recorded. The analysis of the isotherm shapes, supported by Brewster angle microscopy images, allowed to ascertain that reduction of the monolayer area below 0.2 nm^2^ causes the liquid crystal molecules to be pushed out towards the top of the first monolayer being in direct contact with the subphase surface. The model of the molecules arrangement in mixed Langmuir films was proposed.

The Langmuir films were transferred onto the quartz substrates at two surface pressures and mono-and multilayered LB films were formed. For the LB films the electronic absorption spectra under normal incidence of the light were recorded. Analyzing the absorption intensity as well as the transfer ratio of the LB films obtained in various ways, it seems that the best conditions for homeotropic alignment of the fluorinated liquid crystals under investigation can be achieved when the solid substrate is first covered with AA multilayer. The result of such a multilayer was that the TR for deposition of the liquid crystal/AA mixtures, both at low and high pressures, is very close to unity and the results obtained are reliably repeatable. Indeed, in such LB films, the molecules are loosely packed, but usually a looser packing of molecules in command layer allows for some penetration (interdigitation) of the liquid crystal molecules and thus favours their vertical alignment [34]. Another possibility to obtain the homeotropic orientation of the liquid crystal molecules can be the coverage of the solid substrate by one monolayer of the liquid crystal/AA mixture, but then the deposition should take place at the surface pressure before the plateau region.

In further investigations, our conclusions will be checked practically by construction of the VA-type liquid crystal cells for some technologically important mixtures with negative dielectric anisotropy consisting of fluoro-substituted molecules, which properties are reported in [3].

## Figures and Tables

**Table 1.** Features of π-A isotherms of Langmuir films of AA, 8OCFPB, and 8OCFPB/AA mixtures on the water.

		**AA component**				**Liquid crystal component**			

**Compound**	**X_M_**	**A_0_/nm^2^**	**A_C_/nm^2^**	**π_C_/mN·m^−1^**	**C_S_′/m·N^−1^**	**A_0_/nm^2^**	**A_C_/nm^2^**	**π_C_/mN·m^−1^**	**C_S_′/m·N^−1^**
AA	–	0.24	0.19	26.5	7.6	–	–	–	–

8OCFPB/AA	0.1	0.20	0.16	26.9	9.1	0.30	0.24	8.5	20.5
	0.2	0.17	0.14	26.8	8.9	0.36	0.27	8.4	28.5
	0.3	0.15	0.12	26.8	9.9	0.40	0.29	8.2	33.5
	0.4	0.13	0.10	27.1	10.5	0.47	0.32	8.0	38.7
	0.5	–	–	–	–	0.52	0.34	7.9	43.8
	0.6	–	–	–	–	0.57	0.37	7.8	44.9
	0.7	–	–	–	–	0.63	0.39	8.2	47.6
	0.8	–	–	–	–	0.70	0.41	7.9	51.4
	0.9	–	–	–	–	0.73	0.43	7.7	53.4

8OCFPB	–	–	–	–	–	0.72	0.45	7.4	52.1

**Table 2.** Features of π-A isotherms of Langmuir films of N32/AA, N33/AA and N34/AA mixtures on the water.

		**AA component**				**Liquid crystal component**			

**Compound**	**X_M_**	**A_0_/nm^2^**	**A_C_/nm^2^**	**π_C_/mN·m^−1^**	**C_S_′/m·N^−1^**	**A_0_/nm^2^**	**A_C_/nm^2^**	**π_C_/mN·m^−1^**	**C_S_′/m·N^−1^**
N32/AA	0.1	0.21	0.17	26.2	8.5	0.24	0.23	5.5	10.0
	0.2	0.20	0.16	26.2	8.1	0.23	0.22	5.5	10.6
	0.3	0.17	0.14	26.4	8.1	0.18	0.17	5.5	9.0
	0.4	0.16	0.12	26.0	8.1	–	–	–	–
	0.5	0.13	0.10	26.3	8.2	–	–	–	–
	0.6	0.11	0.09	26.6	8.4	–	–	–	–

N33/AA	0.1	0.20	0.17	26.2	8.1	0.25	0.24	2.0	14.4
	0.2	0.19	0.15	26.2	7.9	0.26	0.25	1.9	15.2
	0.3	0.17	0.14	26.4	8.6	0.25	0.24	1.5	19.4
	0.4	0.15	0.12	26.0	9.6	0.21	0.20	1.2	46.9
	0.5	0.13	0.10	26.3	8.7	0.14	0.13	2.1	22.0
	0.6	0.11	0.08	26.6	9.7	–	–	–	–

N34/AA	0.1	0.22	0.18	26.4	7.7	0.26	0.25	1.3	14.2
	0.2	0.19	0.15	26.3	8.8	0.26	0.25	1.4	29.4
	0.3	0.16	0.13	26.0	8.7	0.26	0.25	1.4	23.1
	0.4	0.14	0.11	25.8	8.3	0.28	0.28	1.4	15.5
	0.5	0.12	0.09	26.3	10.0	0.30	0.28	1.4	32.5
	0.6	0.10	0.08	26.4	10.3	0.31	0.30	1.5	23.4

**Table 3.** Features of π-A isotherms of Langmuir films of N52/AA, N53/AA and N54/AA mixtures on the water.

		**AA component**				**Liquid crystal component**			

**Compound**	**X_M_**	**A_0_/nm^2^**	**A_C_/nm^2^**	**π_C_/mN·m^−1^**	**C_S_′/m·N^−1^**	**A_0_/nm^2^**	**A_C_/nm^2^**	**π_C_/mN·m^−1^**	**C_S_′/m·N^−1^**
N52/AA	0.1	0.20	0.16	26.0	9.6	0.24	0.22	7.5	10.0
	0.2	0.17	0.14	26.1	9.9	0.25	0.23	7.7	10.3
	0.3	0.15	0.12	26.5	9.4	0.26	0.24	7.7	10.6
	0.4	0.13	0.11	25.6	9.1	0.27	0.25	7.6	10.6
	0.5	0.11	0.09	25.7	10.8	0.29	0.26	7.4	12.9
	0.6	0.09	0.07	25.4	11.3	0.29	0.26	7.6	12.5

N53/AA	0.1	0.21	0.17	26.2	8.4	0.25	0.23	2.7	20.8
	0.2	0.18	0.15	25.5	8.3	0.26	0.24	2.6	20.9
	0.3	0.15	0.13	25.9	8.4	0.25	0.24	2.6	19.8
	0.4	0.12	0.10	26.0	8.8	0.27	0.26	3.7	13.4
	0.5	0.10	0.08	26.6	9.0	0.28	0.26	3.6	14.2
	0.6	0.09	0.07	25.9	9.0	0.28	0.26	3.3	16.2

N54/AA	0.1	0.21	0.17	26.8	8.2	0.25	0.24	2.7	12.7
	0.2	0.18	0.15	26.8	8.2	0.25	0.25	2.7	10.5
	0.3	0.16	0.13	26.8	8.2	0.29	0.25	2.7	24.6
	0.4	0.15	0.11	26.4	10.4	0.26	0.26	2.6	24.2
	0.5	0.13	0.10	26.4	10.7	0.31	0.28	2.6	25.7
	0.6	0.11	0.08	26.5	11.1	0.32	0.29	2.5	27.0

**Table 4.** Features of π-A isotherms of Langmuir films of AA, 8OCFPB, and 8OCFPB/AA mixtures on CaCl_2_ solution.

		**AA component**				**Liquid crystal component**			

**Compound**	**X_M_**	**A_0_/nm^2^**	**A_C_/nm^2^**	**π_C_/mN·m^−1^**	**C_S_′/m·N^−1^**	**A_0_/nm^2^**	**A_C_/nm^2^**	**π_C_/mN·m^−1^**	**C_S_′/m·N^−1^**
AA	–	0.21	0.18	24.0	6.6	–	–	–	–

8OCFPB/AA	0.1	0.18	0.16	23.3	9.1	0.29	0.23	8.2	22.5
	0.3	0.14	0.12	23.2	10.2	0.40	0.28	8.2	34.4
	0.5	–	–	–	–	0.54	0.35	8.2	42.2
	0.7	–	–	–	–	0.63	0.39	8.3	48.0
	0.9	–	–	–	–	0.78	0.47	8.2	50.0

8OCFPB	–	–	–	–	–	0.74	0.46	7.4	50.9

**Table 5.** Features of π-A isotherms of Langmuir films of N32/AA, N33/AA and N34/AA mixtures on CaCl_2_ solution.

		**AA component**				**Liquid crystal component**			

**Compound**	**X_M_**	**A_0_/nm^2^**	**A_C_/nm^2^**	**π_C_/mN·m^−1^**	**C_S_′/m·N^−1^**	**A_0_/nm^2^**	**A_C_/nm^2^**	**π_C_/mN·m^−1^**	**C_S_′/m·N^−1^**
N32/AA	0.1	0.19	0.17	22.6	7.8	0.24	0.23	5.3	9.5
	0.2	0.18	0.15	22.2	7.8	0.25	0.24	5.5	9.2
	0.3	0.15	0.13	22.6	8.0	0.24	0.23	5.3	9.3
	0.4	0.13	0.11	22.1	8.7	0.24	0.22	4.9	12.6
	0.5	0.11	0.09	22.2	8.9	0.13	0.12	5.4	14.3
	0.6	0.10	0.08	22.1	9.6	–	–	–	–

N33/AA	0.1	0.20	0.17	22.7	7.9	0.24	0.23	2.0	19.0
	0.3	0.16	0.13	22.7	8.3	0.27	0.26	2.0	19.8
	0.5	0.12	0.10	22.4	9.4	–	–	–	–

N34/AA	0.1	0.20	0.16	22.7	7.1	0.24	0.23	1.0	16.4
	0.3	0.16	0.13	22.7	8.1	0.16	0.26	1.0	29.3
	0.5	0.11	0.09	22.5	8.1	0.28	0.28	1.0	22.6

**Table 6.** Features of π-A isotherms of Langmuir films of N52/AA, N53/AA and N54/AA mixtures on CaCl_2_ solution.

		**AA component**				**Liquid crystal component**			

**Compound**	**X_M_**	**A_0_/nm^2^**	**A_C_/nm^2^**	**π_C_/mN·m^−1^**	**C_S_′/m·N^−1^**	**A_0_/nm^2^**	**A_C_/nm^2^**	**π_C_/mN·m^−1^**	**C_S_′/m·N^−1^**
N52/AA	0.1	0.20	0.17	22.7	8.8	0.24	0.23	7.1	8.6
	0.3	0.16	0.13	22.1	9.9	0.26	0.24	7.0	9.4
	0.5	0.11	0.10	22.3	10.1	0.28	0.26	6.9	10.5

N53/AA	0.1	0.20	0.17	22.9	7.7	0.23	0.22	3.7	12.3
	0.3	0.16	0.13	23.2	8.2	0.26	0.24	3.7	14.2
	0.5	0.11	0.09	23.1	9.4	0.27	0.26	3.6	17.3

N54/AA	0.1	0.20	0.17	22.4	7.2	0.23	0.23	1.9	11.3
	0.3	0.16	0.13	22.5	8.2	0.26	0.25	1.9	16.5
	0.5	0.11	0.09	22.6	8.0	0.28	0.27	1.9	14.8
